# Biogenic polymer-based heart valve for congenital cardiac surgery

**DOI:** 10.1016/j.xjon.2025.11.013

**Published:** 2025-11-21

**Authors:** Julian Hubrich, Christopher Herz, Dario Arcuti, Alexandra Zorin, Emma Richert, Stefan Simon, Dominik Obrist, Christian Hagl, Petra Mela, Jürgen Hörer, Thierry Carrel, Jacobus Theron, Linda Grefen, Maximilian Grab, Paul Philipp Heinisch

**Affiliations:** a Department of Congenital and Pediatric Cardiac Surgery, German Heart Center Munich, Technical University, Munich, Germany; b Division of Congenital and Pediatric Cardiac Surgery, LMU Munich University Hospital, Munich, Germany; c EKHZ, European Pediatric Heart Center, Munich, Bavaria, Germany; d German Center for Cardiovascular Research (DZHK), Partner Site Munich Heart Alliance, Munich, Germany; e Department of Cardiac Surgery, LMU Munich University Hospital, Munich, Germany; f Chair of Medical Materials and Implants, Technical University, Munich, Germany; g Cardiovascular Engineering, ARTORG Center for Biomedical Engineering Research, University of Bern, Bern, Switzerland; h Division of Cardiothoracic Surgery, University of Cape Town, Cape Town, South Africa

**Keywords:** congenital heart disease, biogenic polymer-based heart valve, bacterial cellulose

## Abstract

**Background:**

Current heart valve prostheses in congenital cardiac surgery (CCS) are unable to grow, remodel, or adapt to a child's evolving physiology, resulting in increased mortality rates due to material-related limitations. The excellent biocompatibility and hemocompatibility of bacterial cellulose (BC) make it a promising alternative. This study aimed to use BC to develop a biogenic polymer-based heart valve and then to assess its hemodynamic performance and long-term durability.

**Methods:**

Heart valve leaflets were produced via a standard BC protocol and compressed to a minimal thickness, and their biomechanical properties were evaluated. Using a customized template, BC leaflets were sutured into a 23-mm stent scaffold. Two prototype series with different leaflet designs were tested in a mock circulatory flow loop model with a flow rate of 5 L/minute at 120/80 mm Hg. Long-term durability was assessed for 10 ± 0.5 million cycles at 120/80 mm Hg, followed by retesting.

**Results:**

BC valve leaflets exhibited a thickness reduction of 94.01% to 0.3 ± 0.11 mm (*P* < .001) while retaining a durability of 100% at 500 mm Hg (n = 23), with a maximum tensile strength of 1.64 ± 0.3 MPa (n = 35). All valves combined (n = 21) displayed a mean transvalvular pressure drop (MTP) of 8.32 ± 1.23 mm Hg, a mean regurgitation fraction (REG) of 10.22 ± 4.42%, and a mean effective orifice area (EOA) of 1.85 ± 0.14 cm^2^, with valves of series 2 showing a lower REG (*P* < .001). Following long-term durability testing, all valves of series 2 (n = 6) remained intact, demonstrating an MTP 9.11 ± 1.13 mm Hg, REG of 9.41 ± 4.25%, and EOA of 1.7 ± 0.1 cm^2^.

**Conclusions:**

The potential of BC for use in CCS was demonstrated by developing a new biogenic polymer-based valve with excellent hemodynamic performance. These results warrant further investigation and development of this biomaterial.

## Graphical Abstract

**Figure:**
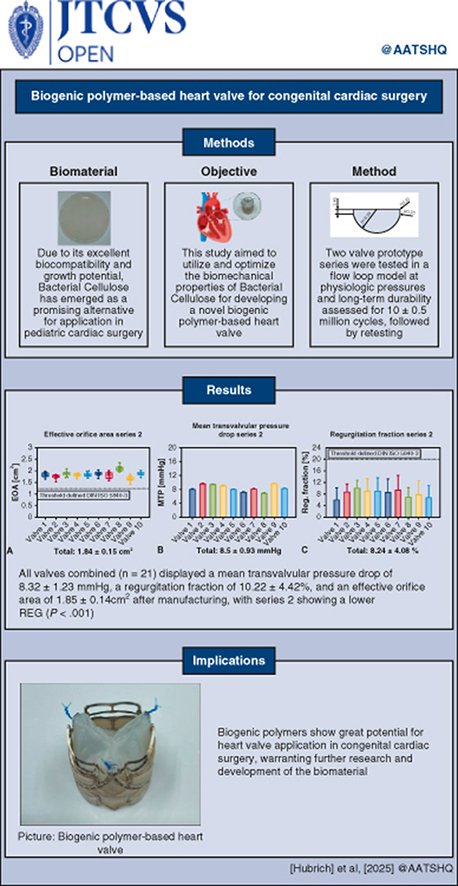

**Figure:**
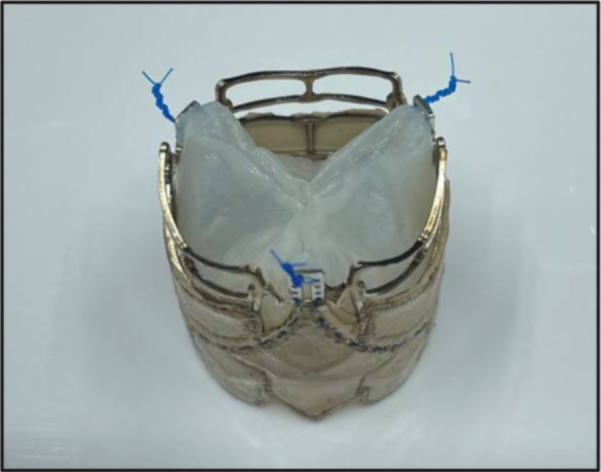
Biogenic polymer-based heart valve.

**Central Message**

Biogenic polymer-based heart valves with bacterial cellulose leaflets demonstrate favorable hemodynamic performance and may hold potential for future growth-adaptive use in pediatric cardiac surgery.

**Perspective**

Bacterial cellulose addresses the limitations of current valve implants in congenital cardiac surgery that are unable to grow or remodel after implantation. By demonstrating enhanced biomechanical resilience and hemodynamic performance, the biogenic polymer-based valve contributes to the ongoing development of materials with the potential to support growth in pediatric valve applications.

Heart valves open and close approximately 70 times per minute, 100,000 times per day, and more than 3 billion times over a lifetime.^1^, ^2^, ^3^ Valvular anomalies account for nearly one-third of newborns with congenital heart disease (CHD), with most cases requiring surgical intervention throughout life.^4^ Despite the increasing number of children with CHD surviving into adulthood, congenital valvular disease remains one of the leading causes of death in pediatric cardiac surgery patients.^5^, ^6^, ^7^ Several strategies for heart valve replacement exist, including mechanical valves, bioprosthetic valves, cryopreserved homografts, and decellularized allografts.^5^^,^^6^

While mechanical heart valves necessitate lifelong anticoagulation, increasing the risk of major bleeding or thromboembolic events, bioprosthetic valves carry a higher risk of early structural degeneration and the need for earlier reinterventions.^5^^,^^8^^,^^9^ However, the primary drawback of all materials is their inability to grow and adapt to a child's evolving physiology after implantation.^10^^,^^11^ Therefore, the current surgical strategy often results in serial reoperations for successively larger implant exchanges, with patients often enduring up to 5 operations in their lifetime, contributing to increased mortality rates.^5^^,^^10^^,^^12^^,^^13^ Thus, there is an urgent need for new innovative approaches for heart valve replacement and repair in children.

Biogenic polymer-based materials, including bacterial cellulose (BC) synthesized by *Acetobacter xylinum*, have emerged as a promising alternative to surmount these material-related limitations. By closely mimicking the human collagen network, this polymer hydrogel facilitates a tissue engineering approach, allowing BC to be colonized and remodeled by endothelial and smooth muscle cells, thereby potentially enabling future growth-adaptive functionality.^14^, ^15^, ^16^, ^17^, ^18^ The excellent biocompatibility and hemocompatibility of BC with cellular colonization has already been demonstrated in large in vivo models with no signs of inflammation or thrombogenicity.^17^^,^^18^ Its high water content of up to 99% contributes to favorable flexibility, resembling cardiac tissue more closely than currently available materials.^15^^,^^16^^,^^19^

Recent studies have improved the biomechanical properties and resilience of BC, making it suitable for further cardiovascular applications.^20^^,^^21^ This study aimed to use BC for prototyping and developing a biogenic polymer-based heart valve and then to assess its hemodynamic performance and long-term durability.

## Materials and Methods

The data supporting the findings of this study are available on reasonable request from the corresponding author. Formal Institutional Review Board/Ethics Review Board approval was not required. Due to the nature of the study, informed consent was not required.

### Production Protocol for Biogenic Polymer-Based Patches and Heart Valve Manufacturing

**Figure 1:**
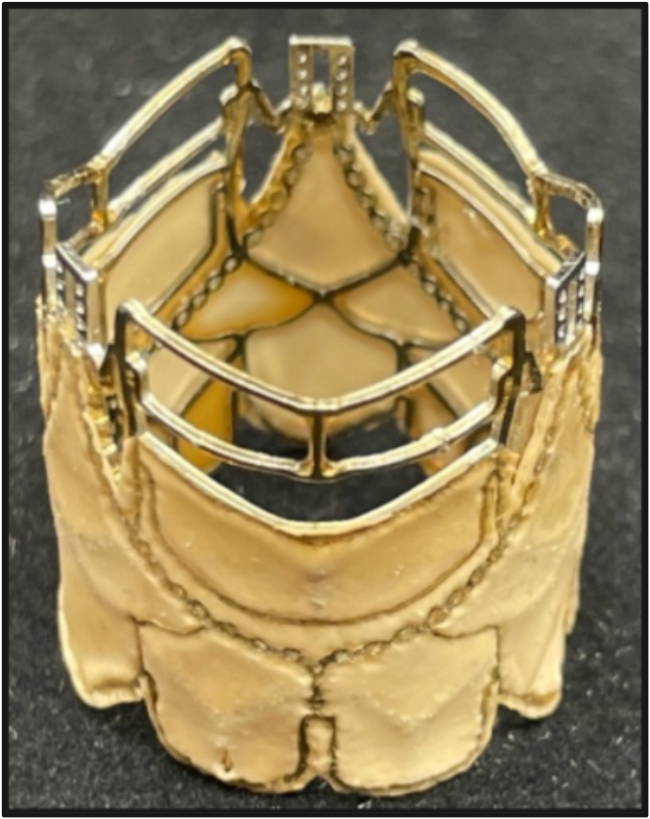
Nickel-cobalt-chromium stent scaffold with stitching holes for valve leaflets.

The standard production protocol of BC patches, as described previously,^22^ and the manufacturing process of the two biogenic polymer-based heart valve prototype series are outlined in the Appendix E1. The biogenic polymer-based heart valve is illustrated in the Central Picture, and a novel nickel-cobalt-chromium stent with stitching holes for bioprosthetic leaflets of an SAT balloon-expandable transcatheter aortic valve replacement (TAVR) valve is shown in Figure 1.^23^

### Biomechanical Characterization of Biogenic Polymer-Based Valve Leaflets

#### Thickness measurement

After compressing the BC patches, samples were used to measure and evaluate the BC valve leaflet thickness at 5 distinct points, including 4 evenly spaced along the outer edge and 1 in the center, allowing for further evaluation of the homogeneity of the material.

#### Inflation pressure testing

For inflation pressure testing BC samples with a diameter of 4.7 cm were secured in a water column with a clamp ring at a tightening torque of 2.5 Nm to prevent lateral movement during pressure application. For recording, a pressure sensor (WIKA A-10 PE81-60, 1.6 bar) and Coolterm software version 1.9.1 (build 964, 64 bit, x86, Roger Meier) were used. Patches were tested at a pressure >500 mm Hg for a minimum duration of 1 minute. Rupture was defined as visible damage of BC corresponding to a step pressure drop in the Coolterm recording.

#### Uniaxial tensile testing

All experiments were conducted with the tensile testing machine (zwickiLine, 2.5 kN; ZwickRoell GmbH) and recorded with the corresponding software (testXpert V12.3; ZwickRoell GmbH). BC samples in a standardized dogbone shape (DIN 53504–S3) were fabricated and clamped for tensile testing. Uniaxial strain stress testing was carried out to evaluate the maximum tensile strength and the elongation at break.

### Hemodynamic Performance Testing of Biogenic Polymer-Based Valves

The biogenic polymer-based valves were tested in a mock circulation system under aortic conditions according to ISO 5840-3: cardiac output of 5 L/minute, aortic pressure of 120/80 mm Hg, and frequency of 70 bpm. The pressures were measured by pressure transducers (CODAN pvd Critical Care GmbH) positioned immediately upstream and downstream from the valve. Instantaneous flow was measured by a flow meter (CO.55/300H V2.0; SONOTEC Ultraschallsensorik Halle GmbH) positioned immediately upstream from the valve. Pressure and flow values were recorded by a LabVIEW application.

**Figure 2:**
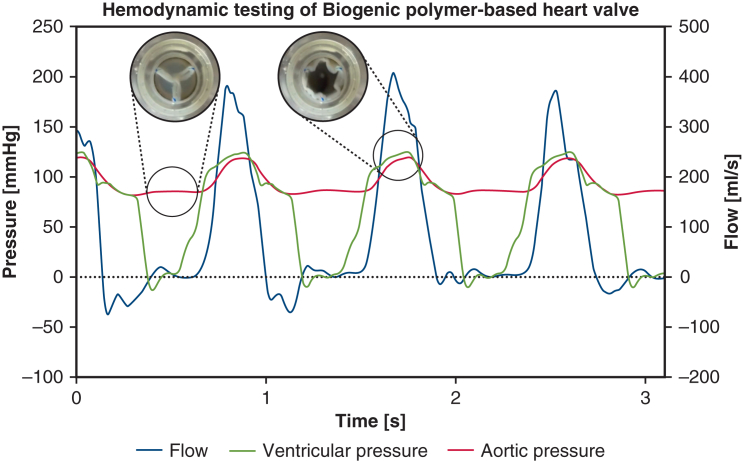
Pressure-flow-time diagram of a biogenic polymer-based heart valve in a mock circulatory flow loop, with images depicting the valve at diastolic closure and systolic opening. Created in BioRender. Hubrich, J. (2026) https://BioRender.com/x6emt9.

In accordance with DIN EN ISO 5840-1, the recorded pressure and flow data of 10 cycles were used to calculate the effective orifice area (EOA), the regurgitant volume, the regurgitant fraction (REG), and the mean transvalvular pressure drop (MTP). The EOA is calculated by:

$$EOA\;\left[{cm}^{2}\right]=\frac{q_{RMS}}{51,6\sqrt{\frac{\Delta P_{mean}}{\rho }}}$$

where q_RMS_ is the root mean square of the forward flow during the positive differential pressure period in mL s^−1^, ΔP_mean_ is the mean pressure difference in mm Hg, and is the density of the test fluid in g cm^−3^ (for water, 1 g cm^−3^). The heart valve ISO 5840-3 defines a regurgitant fraction threshold of 20% and a minimum EOA of 1.25 cm^2^ for a 23-mm transcatheter valve substitute. A pressure-flow-time diagram of a biogenic polymer-based heart valve in a mock circulatory flow loop is shown in Figure 2.

### Long-Term Durability Testing of Biogenic Polymer-Based Valves

After initial performance testing, the last 6 manufactured valves of each series were selected for long-term fatigue cylinder testing. A modified high-cycler (HC) system (HiCycle Durability Tester; ViVitro Labs) was used for all experiments. Valves were placed in the 6 HC chambers, filled with distilled water and antibiotic/antimycotic solution, and tested at physiologic pressure of 120/80 mm Hg for 10 ± 0.5 million cycles, with a visual evaluation every 24 hours. After completion of the durability testing, the valves were inspected and retested in the initial flow loop setup under identical conditions.

### Statistical Analysis

Statistical data analysis was performed using R version 4.4.2 (R Foundation for Statistical Computing). Descriptive analysis included mean ± standard deviation and median with interquartile range. The confidence interval was calculated at 95%. The Shapiro-Wilk test was carried out to determine normality, and the F-test was used for homogeneity of variances. For group comparisons, the *t* test or Wilcoxon rank-sum test was performed. Between-series comparisons used the full observation set for each series, with effect sizes computed from valve-level means with Cohen d. *P* < .05 was considered significant with α = 0.05. Data were visualized in R and BioRender.

## Results

### Biomechanical Characterization of Biogenic Polymer-Based Valve Leaflets

BC heart valve leaflets demonstrated a significant reduction in mean thickness (*P* < .001) of 0.3 ± 0.11 mm (0.28-0.33 mm) after unidirectional compression compared to their thickness of 5.07 ± 0.49 mm (4.94-5.19 mm) at the end of the production process. The average percentage reduction in thickness following compression was 94.01%. In the inflation pressure testing, 100% (n = 23) of the compressed BC patches reached and maintained a pressure >500 mm Hg. The leaflets displayed a mean maximum tensile strength of 1.64 ± 0.3 MPa (1.53-1.74 MPa) (n = 35) and a mean of 19.04 ± 5.14% (17.28%-20.81%) for the elongation at break (n = 35).

### Hemodynamic Performance Testing of Biogenic Polymer-Based Valves

#### Hemodynamic performance of biogenic polymer-based valves after manufacturing

**Figure 3:**
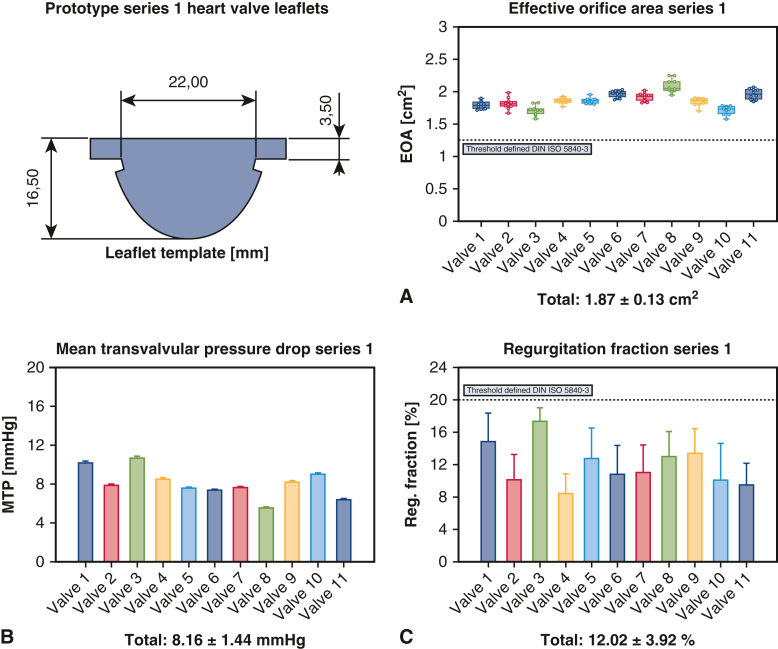
Biogenic polymer-based heart valve prototype series 1 after manufacturing with leaflet template and hemodynamic performance outcomes (A) Effective orifice area (B) Mean transvalvular pressure drop (C) Regurgitation fraction. Box-and-whisker plots show the interquartile range (box, 25th-75th percentiles). The *horizontal line* within each *box* marks the median (50th percentile), and whiskers extend to the observed minimum and maximum values. Created in BioRender. Hubrich, J. (2026) https://BioRender.com/wqofoda.

**Figure 4:**
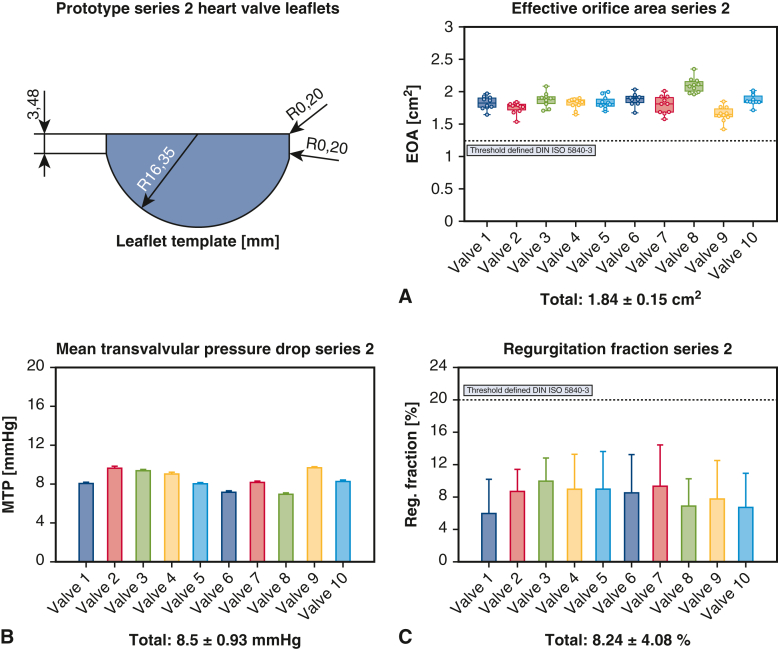
Biogenic polymer-based heart valve prototype series 2 after manufacturing with leaflet template with hemodynamic performance outcomes (A) Effective orifice area (B) Mean transvalvular pressure drop (C) Regurgitation fraction. Box-and-whisker plots show the interquartile range (box, 25th-75th percentiles). The *horizontal line* within each box marks the median (50th percentile), and whiskers extend to the observed minimum and maximum values. Created in BioRender. Hubrich, J. (2026) https://BioRender.com/dfep7a4.

The prototype series 1 (n = 11) exhibited a mean MTP of 8.16 ± 1.44 mm Hg (7.89-8.43 mm Hg), a mean REG of 12.02 ± 3.92% (11.28%-12.76%), and a mean EOA of 1.87 ± 0.13 cm^2^ (1.84-1.89 cm^2^). Prototype series 2 (n = 10) had a mean MTP of 8.5 ± 0.93 mm Hg (8.31-8.68 mm Hg), a mean REG of 8.24 ± 4.08% (7.43%-9.05%), and a mean EOA of 1.84 ± 0.15 cm^2^ (1.81-1.87 cm^2^). In the comparison between the prototype series, a significant difference was observed for MTP (*P* < .05) and REG (*P* < .001), while EOA showed no significant difference (*P* = .132). The effect size for MTP was Cohen d = −0.26 (−1.18 to 0.65), and for REG, Cohen d = 1.8 (0.72-2.88). All valves combined (n = 21) had a mean MTP of 8.32 ± 1.23 mm Hg (8.15-8.49 mm Hg), a mean REG of 10.22 ± 4.42% (9.62%-10.82%) and a mean EOA of 1.85 ± 0.14 cm^2^ (1.84-1.87 cm^2^). The hemodynamic performance of the biogenic polymer-based heart valves after manufacturing is visualized in Figures 3 and 4.

#### Hemodynamic performance of biogenic polymer-based valves before durability testing

The last 6 valves produced per prototype series were selected for initial durability testing. Between the prototype series before durability testing, a significant difference was observed for MTP (*P* < .01), REG (*P* < .001), and EOA (*P* < .05). All valves combined (n = 12) exhibited a mean MTP of 7.77 ± 1.08 mm Hg (7.57-7.96 mm Hg), a mean REG of 9.74 ± 4.27% (8.97%-10.51%), and a mean EOA of 1.89 ± 0.16 cm^2^ (1.86-1.91 cm^2^). The final 6 valves of each series demonstrated enhanced hemodynamic performance compared to the initial valves of the same prototype series, both for series 1, with significant differences in MTP (*P* < .001) and EOA (*P* < .001) but no significant difference in REG (*P* = .129), and series 2, with a significant difference for MTP (*P* < .001) but no significant differences in EOA (*P* = .135) and REG (*P* = .664).

#### Hemodynamic performance of biogenic polymer-based valves after durability testing

**Figure 5:**
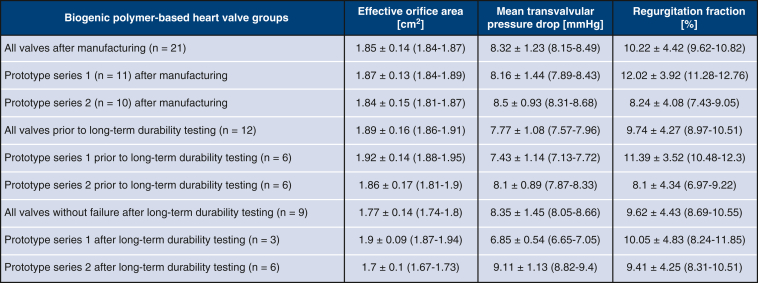
Hemodynamic performance of biogenic polymer-based heart valve groups. Created in BioRender. Hubrich, J. (2026) https://BioRender.com/ohn1oc5.

After long-term durability testing, the valves were retested under identical conditions. For prototype series 1, 3 valves failed during the fatigue cylinder testing, with a failure mechanism on the commissures and visual damage to the valve. The remaining valves of the series (n = 3) had a mean MTP of 6.85 ± 0.54 mm Hg (6.65-7.05 mm Hg), a mean REG of 10.05 ± 4.83% (8.24%-11.85%), and a mean EOA of 1.9 ± 0.09 cm^2^ (1.87-1.94 cm^2^). All valves of prototype series 2 (n = 6) remained intact during long-term HC testing, with a mean MTP of 9.11 ± 1.13 mm Hg (8.82-9.4 mm Hg), a mean REG of 9.41 ± 4.25% (8.31%-10.51%), and a mean EOA of 1.7 ± 0.1 cm^2^ (1.67-1.73 cm^2^). Compared to the individual test series before durability testing, valve prototype series 1 exhibited a significant difference in MTP (*P* < .001) but no significant differences in REG (*P* = .182) and EOA (*P* = .624). For series 2, there was no significant difference in REG (*P* = .097) but significant differences in EOA (*P* < .001) and MTP (*P* < .001) were seen. All remaining valves after durability testing combined displayed a mean MTP of 8.35 ± 1.45 mm Hg (8.05-8.66 mm Hg), a mean REG of 9.62 ± 4.43% (8.69%-10.55%), and a mean EOA of 1.77 ± 0.14 cm^2^ (1.74-1.8 cm^2^). All valves combined after the manufacturing process, compared to the remaining valves after durability testing, only showed a significant difference only for EOA (*P* < .001), with no significant difference for REG (*P* = .292) or MTP (*P* = .871). Performance data are illustrated in Figure 5.

Therefore, all valves from both prototype series not only met but exceeded the in vitro hydrodynamic device performance requirements for 23-mm aortic valves (DIN ISO 5840-3) after manufacturing for EOA and REG. Following durability testing for 10 ± 0.5 million cycles, all valves of prototype series 2 still surpassed an EOA of 1.25 cm^2^ and REG <20%.

## Discussion

### Limitations of Current Heart Valve Implants in Congenital Cardiac Surgery and the Emerging Potential of BC

All current heart valve implants in congenital cardiac surgery face limitations, resulting in higher mortality and morbidity rates due to material-related limitations.^5^^,^^7^^,^^24^ The primary drawback of all valve implants is their inability to grow, remodel, or adapt to a child's evolving physiology after implantation, underscoring the need for new heart valve replacement and repair approaches in children.^10^^,^^11^

BC, a biogenic polymer-based material synthesized by *Acetobacter xylinum*, has recently been identified as a viable option for addressing these material-related constraints.^21^ This polymer hydrogel closely resembles the human collagen network, enabling a tissue engineering approach and allowing for colonization and remodeling of BC by endothelial and smooth muscle cells.^14^, ^15^, ^16^, ^17^, ^18^ This may make BC suitable for potential future growth-adaptive applications.

The biomechanical properties and durability of BC have been improved recently through the refinement of growth media compositions, making it suitable for further cardiovascular applications.^20^^,^^22^ In this study, we aimed to optimize and use BC for the development of a novel biogenic polymer-based heart valve and assess its hemodynamic performance and long-term durability. Two prototype series with different compressed BC leaflet designs were evaluated in a mock circulatory flow loop setup under physiologic conditions, and long-term durability was assessed.

### Enhancement of Biomechanical Properties of BC

By unidirectionally compressing BC patches, the biomechanical properties of the valve leaflets were enhanced significantly. In a recent feasibility study, only 1 BC patch and 1 standard xenograft pericardial patch achieved a pressure of 200 mm Hg, whereas the remaining BC patches ruptured at lower pressures.^20^ In contrast, the BC heart valve leaflets could consistently and reproducibly reach and maintain pressures >500 mm Hg, with a durability rate of 100% (n = 23). The maximum tensile strength surpassed previously reported strains of the genus *Komagataeibacter* at 0.68 ± 0.13 MPa, compared to our BC leaflets with 1.64 ± 0.3 MPa (n = 35).^25^ By thickness reduction via compression, BC, which consists of up to 99% water, primarily loses fluid.^19^ This may increase the relative proportion of cellulose nanofibers in the leaflet, thereby enhancing biomechanical resilience and achieving high batch-to-batch reproducibility as essential prerequisites for its use as a heart valve leaflet.

### Hemodynamic Performance Testing of Biogenic Polymer-Based Valves

For evaluating hemodynamic performance, 3 key metrics were identified: EOA, MTP, and REG. According to DIN ISO 5840-3 for aortic valve substitutes implanted by transcatheter techniques, the minimum requirement is an EOA of 1.25 cm^2^ and REG <20% for a 23-mm stent. BC valves were compared to these requirements; valves were not crimped prior to flow loop testing. Both prototype series not only met but exceeded the in vitro hemodynamic performance requirements for EOA and REG, with all valves combined (n = 21) exhibiting an MTP of 8.32 mm Hg (8.15-8.49 mm Hg), a REG of 10.22% (9.62%-10.82%), and an EOA of 1.85 cm^2^ (1.84-1.87 cm^2^). In a direct comparison, series 2 demonstrated a lower REG (*P* < .001) and better hemodynamic performance. The rationale for this discrepancy may lie in the different leaflet designs, with prototype 1 having a smaller leaflet size, potentially leading to a less complete valve closure and thus an increased REG. The surgical implantation technique used in prototype series 2, placing stitches in a closer, evenly tight interval at the commissure, also may have increased stability and reduced possible leakage.

In clinical practice, hemodynamic trends from postimplantation to 3 months were demonstrated for transfemoral TAVR, showing a mean gradient of 12.5 (12.0-12.9) mm Hg to 9.7 (9.50-9.91) mm Hg and EOA of 1.65 (1.62-1.67) cm^2^ to 1.71 (1.67-1.77) cm^2^, and for surgical aortic valve replacement, with a mean gradient of 10.3 (5.44-18.9) mm Hg to 10.8 (10.2-11.4) mm Hg, and EOA of 1.46 (1.40-1.50) cm^2^ to 1.64 (1.44-1.88) cm^2^.^26^ A mean aortic regurgitation fraction of 13.0 ± 9.6% was determined by cardiac magnetic resonance after transcatheter aortic valve implantation.^27^ In comparison, our prototype series 2 (n = 10) exhibited a lower mean MTP of 8.5 (8.31-8.68) mm Hg, a higher EOA of 1.84 (1.81-1.87) cm^2^ and a reduced REG of 8.24% (7.43%-9.05%), demonstrating the high potential of biogenic polymer-based materials for heart valve application and development. High reproducibility of the valve manufacturing process (n = 21) and hemodynamic performance were demonstrated, with no individual valve failing to surpass DIN ISO 5840-3 in vitro minimal requirements for EOA and REG.

### Durability Testing of Biogenic Polymer-Based Valves

In fatigue cylinder testing, prototype series 2 demonstrated superior long-term durability, with all valves of series 2 continuing to surpass the in vitro requirements of an EOA of 1.25 cm^2^ and REG <20%. Series 1 was designed to allow for enhanced leaflet movement through a specific leaflet layout and surgical implantation technique, but this may have led to faster wear of the material and earlier failure on the commissures. The series also exhibited a higher REG prior to HC testing (*P* < .001); this increased regurgitation flow with each valve closure might have further increased the susceptibility to early failure. In clinical practice, a reduction of the aortic valve mean gradient and an increase of the EOA of up to 0.09 cm^2^ in the first months postimplantation were observed in the longitudinal analysis for TAVR after implantation.^26^ In the hemodynamic analysis, our valves tended to show a decrease in performance, particularly in EOA. This may be attributable to the experimental setup. The durability testing of BC with distilled water may have led to fluid shifts into BC owing to its higher osmotic pressure, resulting in increased leaflet thickness, leading to a decrease in EOA and affecting the hemodynamic performance. This effect might not be present in blood with higher osmotic pressure, necessitating further investigation into the interaction of BC and blood in vivo. Despite this potential effect, the EOA in series 2 with an EOA of 1.7 (1.67-1.73) cm^2^ still surpassed the discussed minimum in vitro requirement of 1.25 cm^2^. The results of this study carry significant implications for the clinical translation of BC, demonstrating the potential to optimize and use BC for application as a biogenic polymer-based heart valve in congenital cardiac surgery.

### Limitations

One limitation of our study is that all experiments were conducted in vitro, and we cannot report any in vivo data from the biogenic polymer-based heart valve. The excellent biocompatibility and hemocompatibility of BC has been demonstrated previously^17^; however, this also needs to be shown for our BC patches and leaflets. This might require additional production steps, such as autoclaving the material after leaflet implantation. Furthermore, this study was not designed to assess the growth of the biogenic polymer-based heart valves. While BC showed favorable characteristics in the past, including excellent biocompatibility, structural remodeling, cellular integration, and neovascularization, its capacity to support growth, particularly in the context of valve leaflets, remains unproven.^14^, ^15^, ^16^, ^17^, ^18^ Demonstrating such behavior would require dedicated long-term studies in growth-capable large animal models, which are beyond the scope of the current investigation.

While the 23-mm stent may be advantageous in the current development stage for focusing on the hemodynamic performance of the BC leaflets, it would be beneficial for clinical application to demonstrate the possibility of crimping the valves. Developing a cellulose scaffold for leaflet implantation also would also enable construction of a heart valve entirely of BC, meriting further investigation of this biomaterial.

## Conclusions

**Figure 6:**
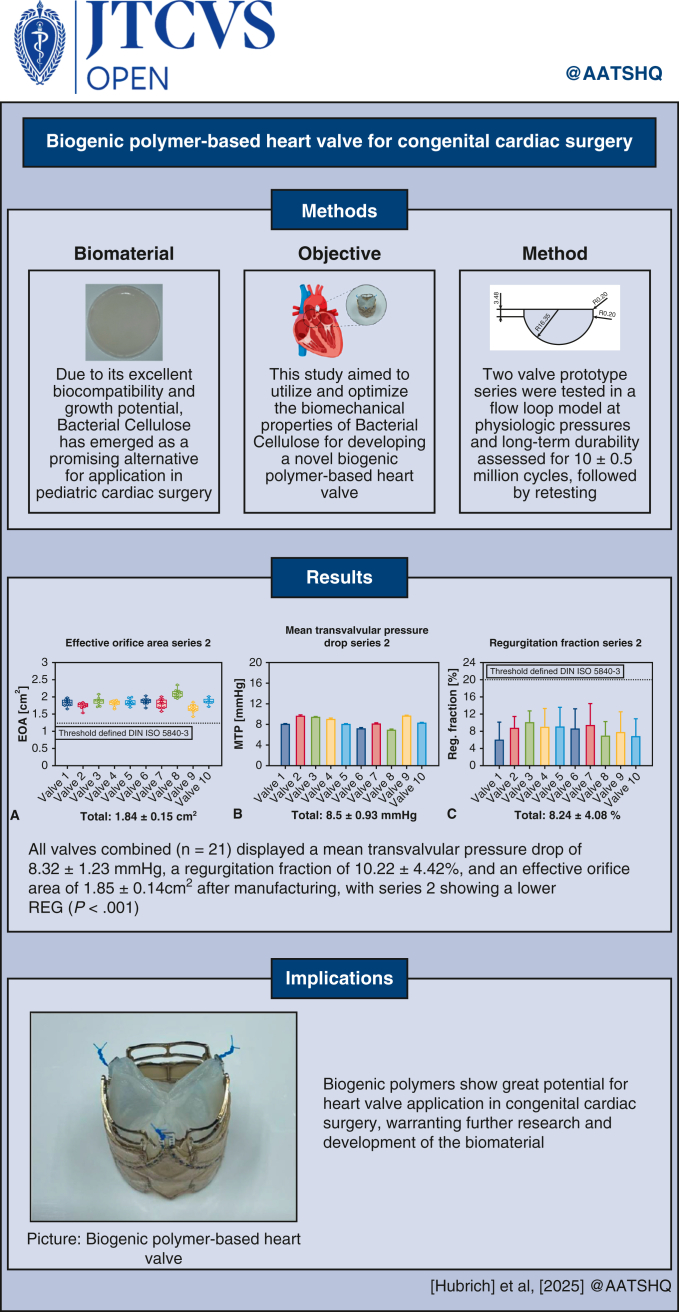
(Graphical abstract). Biogenic polymer-based heart valves exhibit excellent hemodynamic performance and long-term durability. Created in BioRender. Hubrich, J. (2026) https://BioRender.com/7675pqh.

Our study demonstrates that BC can be optimized and used as a heart valve leaflet, retaining high biomechanical resilience after unidirectional compression. Two prototype series of a novel biogenic polymer-based heart valve exhibited a combined (n = 21) MTP of 8.32 (8.15-8.49) mm Hg, REG of 10.22% (9.62%-10.82%), and EOA of 1.85 (1.84-1.87) cm^2^ in a mock circulatory flow loop model, with series 2 showing more favorable performance. Following long-term durability testing, all valves of series 2 remained intact with excellent hemodynamic performance. Thus, biogenic polymers show great potential for heart valve application in congenital cardiac surgery, warranting further research and development of the biomaterial (Figure 6).

## Conflict of Interest Statement

The authors reported no conflicts of interest.

The *Journal* policy requires editors and reviewers to disclose conflicts of interest and to decline handling or reviewing manuscripts for which they may have a conflict of interest. The editors and reviewers of this article have no conflicts of interest.

## APPENDIX E1. Production Protocol for Biogenic Polymer-Based Patches and Heart Valve Manufacturing

### Bacterial Strain

For all experiments, the bacterial strain *Komagateibacter sucrofermentans* (DSM lot no. 15973-0821-001, DSMZ-German Collection of Microorganisms and Cell Cultures) was used. The bacteria were cultivated at a constant temperature of 30 °C in a static and aerobic environment, with an ATCC media exchange of 10 mL every 48 to 72 hours.

### Standard Production Protocol of Biogenic Polymer-Based Patches

BC patches were produced according to a standard production protocol to ensure the highest biomechanical resilience.^22^ The production encompassed an initial dynamic incubation phase of homogenized BC combined with C-Medium at 30 °C with 220 rpm for 72 hours in a 500 mL Erlenmeyer flask, followed by a customized filtration process including centrifugation twice for 5 minutes at 4000 rpm and optical density measurement carried out to ensure batch-to-batch reproducibility. For the final production step, 2 mL of the bacteria solution and 198 mL of growth media, consisting of ATCC-Medium, C-Medium, and 0.9% NaCl, were prepared and incubated statically for 168 hours at 30 °C. During the postproduction, patches were boiled at 80 °C for 30 minutes each in distilled water and a NaOH solution. The patches were stored in 0.9% NaCl at 4 °C. For the application of BC as heart valve leaflets, the patches were compressed unidirectionally at the end of the production process and then biomechanically characterized.

### Manufacturing of Biogenic Polymer-Based Heart Valves

Heart valves were manufactured with biogenic polymer-based patches using custom 3D-printed leaflet templates. For all presented prototype series, a novel nickel-cobalt-chromium stent with stitching holes for bioprosthetic leaflets of an SAT balloon-expandable TAVR valve was used.^23^ The continuous scallop design offers an optimal attachment of valve leaflets and was designed to enhance durability in younger patients.^23^ In this study, 2 different leaflet designs and surgical implantation strategies were used and evaluated. The leaflets were implanted sequentially into the stent. First, sutures were placed at distance between the stent and the leaflet, followed by parachuting the BC leaflets down. First, one side was sutured with 5-0 polypropylene from the nadir to the commissure, followed by the opposite side. The process was replicated with the following leaflet while keeping all sutures under tension. Continuous rehydration of the leaflets was ensured throughout the process, and knots were secured after implantation of the third leaflet. In prototype series 1 (n = 11), the sutures were placed tightly in the prefabricated stent holes at the nadir and widened toward the commissure to allow for enhanced leaflet movement. In prototype series 2 (n = 10), all sutures were placed as tightly as possible, ranging from the nadir to the commissure, by stitching through each stent hole individually. To minimize the risk of leakage at the commissures, the final stitch of each leaflet was placed through the highest suture hole in the stent frame and then tied to the suture from the adjacent leaflet, thereby reducing potential leakage at the leaflet junctions. The biogenic polymer-based heart valve is illustrated in the Central Picture, and the stent scaffold is shown in Figure 1.
